# A Machine Learning-Based Model to Predict In-Hospital Mortality of Lung Cancer Patients: A Population-Based Study of 523,959 Cases

**DOI:** 10.3390/arm91040025

**Published:** 2023-08-09

**Authors:** Que N. N. Tran, Minh-Khang Le, Tetsuo Kondo, Takeshi Moriguchi

**Affiliations:** 1 Emergency & Critical Care Medicine Department, Graduate School of Medicine, Faculty of Medicine, University of Yamanashi, Yamanashi Prefecture, 1110 Shimokato, Chuo City 409-3898, Japan; tmoriguchi@yamanashi.ac.jp; 2 Pathology Department, Graduate School of Medicine, Faculty of Medicine, University of Yamanashi, Yamanashi Prefecture, 1110 Shimokato, Chuo City 409-3898, Japan

**Keywords:** lung cancer, in-hospital mortality, model training, risk stratification, patient-centered strategy

## Abstract

**Highlights:**

**What are the main findings?**

- Our model including six epidemiological components was successfully validated on both internal and external validation.
- Risk stratification by the model showed significantly different survival patterns even after discharge.
- Three well-developed interfaces are friendly to both physicians and patients for prognosis-related conversations.

**What is the implication of the main finding?**

- Our model with easily accessible variables showed its robustness in inferring its predictive value with respect to in-hospital mortality of lung cancer patients.
- The model is highly applicable in follow-up.
- Its applications are useful to clinical in the assistance of strategic planning and the improvement of end-of-life care.

**Abstract:**

**Background:** Stratify new lung cancer patients based on the risk of in-hospital mortality rate after diagnosis. **Methods:** 522,941 lung cancer cases with available data on the Surveillance, Epidemiology, and End Results (SEER) were analyzed for the predicted probability based on six fundamental variables including age, gender, tumor size, T, N, and AJCC stages. The patients were randomly assigned to the training (*n* = 115,145) and validation datasets (*n* = 13,017). The remaining cohort with missing values (*n* = 394,779) was then combined with the primary lung tumour datasets (*n* = 1018) from The Cancer Genome Atlas, Lung Adenocarcinoma and Lung Squamous Cell Carcinoma projects (TCGA-LUAD & TCGA-LUSC) for external validation and sensitivity analysis. **Results:** Receiver Operating Characteristic (ROC) analyses showed high discriminatory power in the training and internal validation cohorts (Area under the curve [AUC] of 0.78 (95%CI = 0.78–0.79) and 0.78 (95%CI = 0.77–0.79), respectively), whereas that of the model on external validation data was 0.759 (95%CI = 0.757–0.761). We developed a static nomogram, a web app, and a risk table based on a logistic regression model using algorithm-selected variables. **Conclusions:** Our model can stratify lung cancer patients into high- and low-risk of in-hospital mortality to assist clinical further planning.

## 1. Introduction

In 2022, there were approximately two million new cases of lung cancer diagnosed in the U.S. [1]. Corresponding to that, there was about a third of that number accounted for lung cancer deaths in the same year [1]. In comparison to those of GLOBOCAN a decade ago, it can be considered an impressive step forward in lung cancer treatment with the increasing trend of survival [2]. Nevertheless, that is not the case in low and middle-income countries, where lung cancer remains challenging as the mortality rate is approximately ninety percent of the incidence rate [3]. Thus, it cannot be denied that early detection as well as the provision and the approachability of innovative therapies in lung cancer treatment play game changers [1,2,3]. Interestingly, sex can be also reckoned as a key point in response to immunotherapy that unveils the reason for lower mortality rate in women than their counterparts, especially in developed nations [3].

For decades, cutting-edge studies in lung cancer treatment focusing on molecular insights, especially in immune checkpoint inhibitors, have resulted in remarkable findings [4]. Whilst immunotherapy interventions have achieved a significant increase in survival of non-small cell lung cancer (NSCLC) patients with oncogenic driver negative [4], surgery approaches still keep an unreplaceable role in the standard care for NSCLC in early stages [5]. Nonetheless, to what extent adjuvant or neoadjuvant chemotherapy is considered overweighing its toxicity risk remains questionable [5,6]. In contrast, small cell lung cancer (SCLC) with a higher metastasis pattern cannot catch up with its counterpart in decreasing incidence-based mortality [7]. However, chemoimmunotherapy has recently shown its potential in the maintenance of life quality of extensive-stage SCLS patients [8]. Notably, liabilities of PD-1 axis inhibition in combination with the platinum-based chemotherapy that were approved by The United States Food and Drug Administration (FDA) in 2018 based on the two randomized controlled trials of IMPOWER 133 [9] and CASPIAN [10] leading to the survival improvement.

The scientific development in screening and treatment of lung cancer brings about early detection and lower mortality, whereas it also puts new challenges to healthcare services. On the one side, there is a limit of studies for the estimation of cost-effectiveness to age ranges of lung cancer screening [11,12]. Likewise, although the policymaking of healthcare services varies from country to country, it is important to have a patient-centered outlook. On the flip side, after diagnoses, it is not straightforward to handle the conversations in which physicians and patients discuss about first-line strategies so that it comes to be realistic and supportive not only in disease treatment but also in quality-of-life enhancement [12,13].

There are some researches revealing the correlation between lung cancer and other factors such as sex [14,15], frailty [16], and brain metastasis [17]. Despite that fact, there is no finding to stratify the in-hospital mortality risk of new lung cancer patients at current hospital admissions for diagnoses, only based on minimum epidemiological components. Subsequently, this individualized approach can assist healthcare providers to quantitatively strategize not only disease treatment but also end-of-life care in the first place, especially in countries with less accessible healthcare services. The present study was conducted based on the epidemiology dataset of the Surveillance, Epidemiology, and End Results (SEER) and The Cancer Genome Atlas, Lung Adenocarcinoma and Lung Squamous Cell Carcinoma projects (TCGA-LUAD and TCGA-LUSC) to address those tackles.

## 2. Materials and Methods

### 2.1. Data Processing

**Figure 1:**
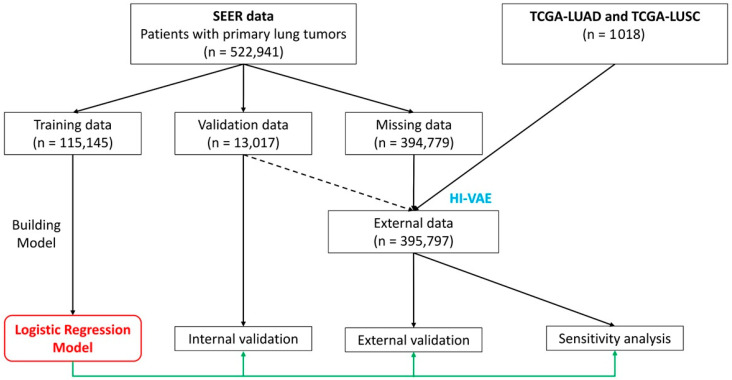
The flowchart shows the study design. SEER data of primary lung cancer patients (*n* = 522,941) is divided into missing data (*n* = 394,779) and fully available data (*n* = 128,162). The fully available data is then randomly assigned to training (*n* = 115,145) and internal validation data (*n* = 13,017). The internal validation data and TCGA data (*n* = 1018) are used to enrich the data to be imputed by heterogenous incomplete variational autoencoder (HI-VAE). After imputation, the internal validation part of the imputed data is excluded. The imputed data is left with only formerly missing data (*n* = 394,779) and TCGA data (*n* = 1018), which is now called external data. The training data is used for model development while validation data and external data are used for internal and external validation. Sensitivity analyses are also performed in the external validation data.

We accessed the SEER program to retrieve information about patients with primary lung tumors. There was a total of 523,941 patients with clinical data, including age, gender, race, tumor size, TNM stages, AJCC stages, overall survival (OS) time, and OS status. We excluded cases with Tis and T0 in the T stage (*n* = 1000). We next defined the selection criteria for the training process, which included patients with the data full availability of age, gender, race, tumor size, TNM stages, and AJCC stages. Finally, 128,162 patients met the criteria and were included in the model training. In the SEER database, the OS time and status were recorded from the time of diagnosis. We randomly assigned the patients to the training (*n* = 115,145) and validation datasets (*n* = 13,017). The remaining cohort with missing values (*n* = 394,779) was then combined with the primary lung tumor datasets from TCGA-LUAD and TCGA-LUSC (Figure 1). Since the data of these 3 datasets are not fully available for the model’s prediction, we performed data imputation by a deep learning technique, using validation data to provide information about missing values.

### 2.2. Defining In-Hospital Mortality

We defined in-hospital mortality in SEER as (1) OS time is ≤ 1 month after diagnosis and (2) OS status is death. Patients without OS time or OS status were excluded from the analysis. Of note, patients who passed after 1 month (OS time > 1 month) were considered to have no in-hospital mortality. Although the definition is not entirely correct because of the complexity of the clinical contexts, we believe that it describes appropriately in most cases. According to previous studies, the median length of stay of lung cancer patients who passed during the first hospital stay was 18.5 days [18] and in-hospital mortality was defined as death within 28 days of admission [19].

### 2.3. Model Training

We divided the training process into 3 phases: statistical selection, information selection, and model fitting. The statistical selection phase included univariate and multivariate logistic regression analyses of interested variables with regard to in-hospital mortality. The information selection phase considered the variables with mutual information (such as tumor size, T stage, and AJCC stage) and the reliability of the variables (such as race). The final step is to fit the logistic regression model with selected features, using the default optimization algorithm.

### 2.4. Model Evaluation

The model evaluation section consisted of 3 parts, (1) internal validation, (2) external validation with data imputation, and (3) sensitivity analysis. In internal validation, we performed receiver operating characteristics (ROC) analysis and bootstrap-estimated calibration in both training data and validation data. In external validation, we combined the TCGA-LUAD and TCGA-LUSC. The necessary variables in the external cohorts were used and missing values were imputed by heterogeneous incomplete variational auto-encoder (HI-VAE) (Supplementary Figure S1). In sensitivity analysis, we employed the survSens package to examine the robustness of the model with respect to the stratification of prognosis in addition to in-hospital mortality.

### 2.5. Analysis Platform

Descriptive statistics of continuous and categorical variables were median (range) and the number of cases (percentage). We used Wilcoxon and chi-square tests to compare the difference in continuous and categorical features, respectively. The HI-VAE model was trained, using the original pipeline published by Nazabal et al. [20]. Other analyses were performed, using R software version 4.2.2 (The R Foundation, Vienna, Austria).

## 3. Results

### 3.1. Patient Characteristics

We described and compared the clinical features of training (*n* = 115,145), validation (*n* = 13,017), and test (*n* = 395,797) cohorts (Table 1). Overall, many characteristics, including age, gender, race, AJCC stage, T stage, N stage, M stage, surgery, and in-hospital mortality, were significantly different (*p* < 0.001). This was related to the different distribution of test data, regarding all the variables. On the other hand, the tumor size and survival time were not different between the 3 cohorts (*p* = 0.494 and *p* = 0.494).

### 3.2. Model Training

First, we listed the interested variables (predictors), including age, gender, race, tumor size, TNM stages, and AJCC stages. The first phase involved the statistical selection of these variables to build a simpler version of the model. Table 2 shows the univariate and multivariate logistic regression analyses of the predictors. By contrast, multivariate analyses required the full availability of all predictive variables. In univariate analysis, race was not significant in all categories and, therefore, was excluded from further training processes. In multivariate analysis, the odds ratio (OR) of the M stage could not be calculated. This was because AJCC stage IV consisted of M1 tumors (OR = 8.20; 95%CI = 7.58–8.97; *p* < 0.001). Therefore, the M stage was also excluded. In the second phase, we noted that the predictive power of the N2 and N3 stages was not appropriate. N2 stage (OR = 1.23; 95%CI = 1.17–1.30; *p* < 0.001) had higher predictive power of in-hospital mortality than N3 (OR = 1.09; 95%CI = 1.03–1.16; *p* = 0.005). It is possible that there was an unobserved confounder underlying this paradox. Therefore, we clumped N2 and N3 categories into the “N2-N3” category while N0 and N1 stages were assigned to the “N0-N1” category because of their insignificant difference (OR = 1.00; 95%CI = 0.93–1.08; *p* = 0.950) in estimating-hospital mortality (Supplementary Table S1). We then proceeded our training process to the final phase, model fitting. The model predictive variables included age (year), gender, tumor size (cm), T (T1, T2, T3, and T4), N (N0-1 and N2-3), and AJCC (I, II, III, and IV) stages.

### 3.3. Model Evaluation

**Figure 2:**
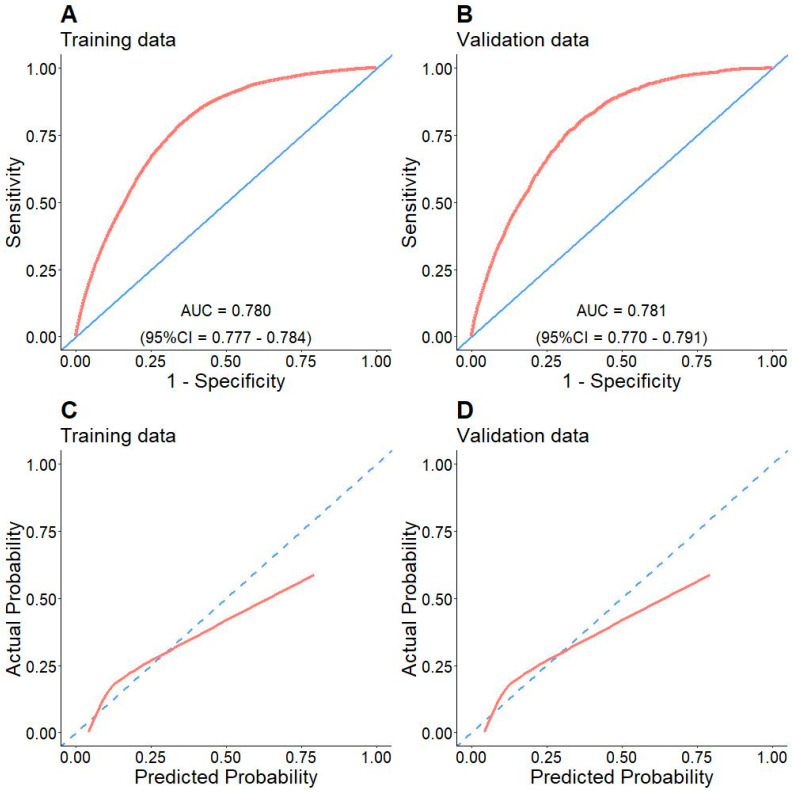
ROC analyses in training (**A**) and validation (**B**) data. The AUCs are 0.780 and 0.781, respectively. The calibration plots in training (**C**) and validation (**D**) shows the concordant and discordant parts between predicted and observed in-hospital mortality probability compared to ideal prediction (dashed line).

**Figure 3:**
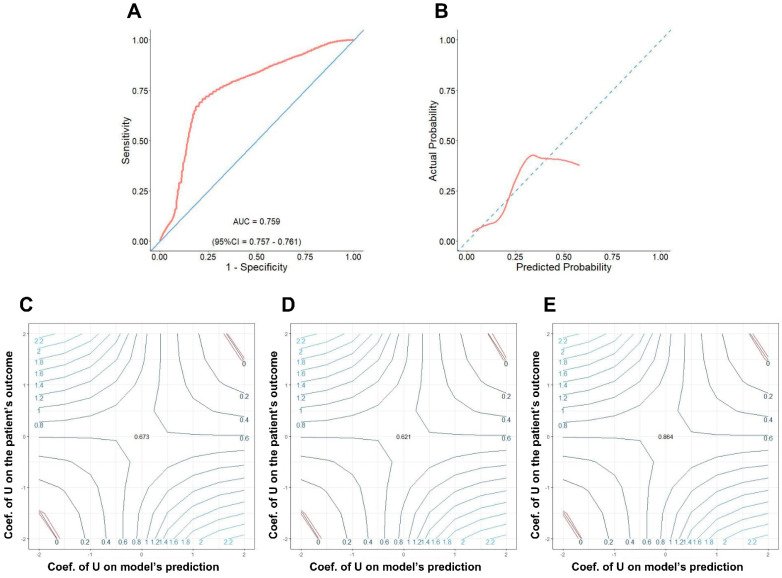
(**A**) ROC analysis and (**B**) calibration plot of the model in external validation cohort. The contour plots (**C**–**E**) show the sensitivity analyses of the model when 25th, 50th, and 75th cut-offs are used. The red contours described the 95% confidence interval of t-statistics (which are described by the original paper of this method), outside of where the model loose its confidence in predicting the patient’s outcome.

In internal validation, we performed ROC analyses on training and validation data. Figure 2A,B shows the ROC curves in training and validation cohorts. The training and validation area under the curve (AUC) were 0.78 (95%CI = 0.78–0.79) and 0.78 (95%CI = 0.77–0.79), respectively. In the calibration plots (Figure 2C,D), the model showed a high concordance between predicted and observed in-hospital mortality by bootstrapping the data. In external validation, we first performed data imputation by HI-VAE. The results of variable-specific errors were reported in Supplementary Table S2. The interpretation of these errors was mentioned in the original paper [20]. The ROC AUC of the model on external validation data was 0.759 (95%CI = 0.757–0.761, Figure 3A) while the calibration plot (Figure 3B) showed a fair performance. From a probability of 0 to 50% of in-hospital mortality, the model estimated probability was fairly in concordance with the observed one while it was mis-calibrated when the true probability was higher than 50%. To further validate the robustness of the model, we performed sensitivity analyses to investigate whether the model prediction remained prognostic under the presence of an unobserved confounder. We treated the model’s prediction as a binary treatment similar to the original paper with three different cut-offs at the 25th, 50th, and 75th values. We adjusted the sensitivity parameters ζ_Z_ and ζ_T_ from −2 to 2 with 0.5 increments. The contour plots were drawn to examine the robustness of the model’s prediction in external dataset. This analysis pipeline was related to the methodology of our previous study [21]. The interpretation was that the model prediction was still meaningful unless there was a presence of the strong unobserved confounder affecting both the patient’s outcome (ζ_T_) and the model’s prediction (ζ_Z_). The red contours enclosed the area of sensitivity parameters where the model still retained its confidence in terms of prognosis. In the present study, the model prognostic value remained informative for the most of the ζ_Z_ and ζ_T_ values in the 3 contour plots (Figure 3C–E), which was a good sign of robustness.

### 3.4. Risk Stratification by the Model

**Figure 4:**
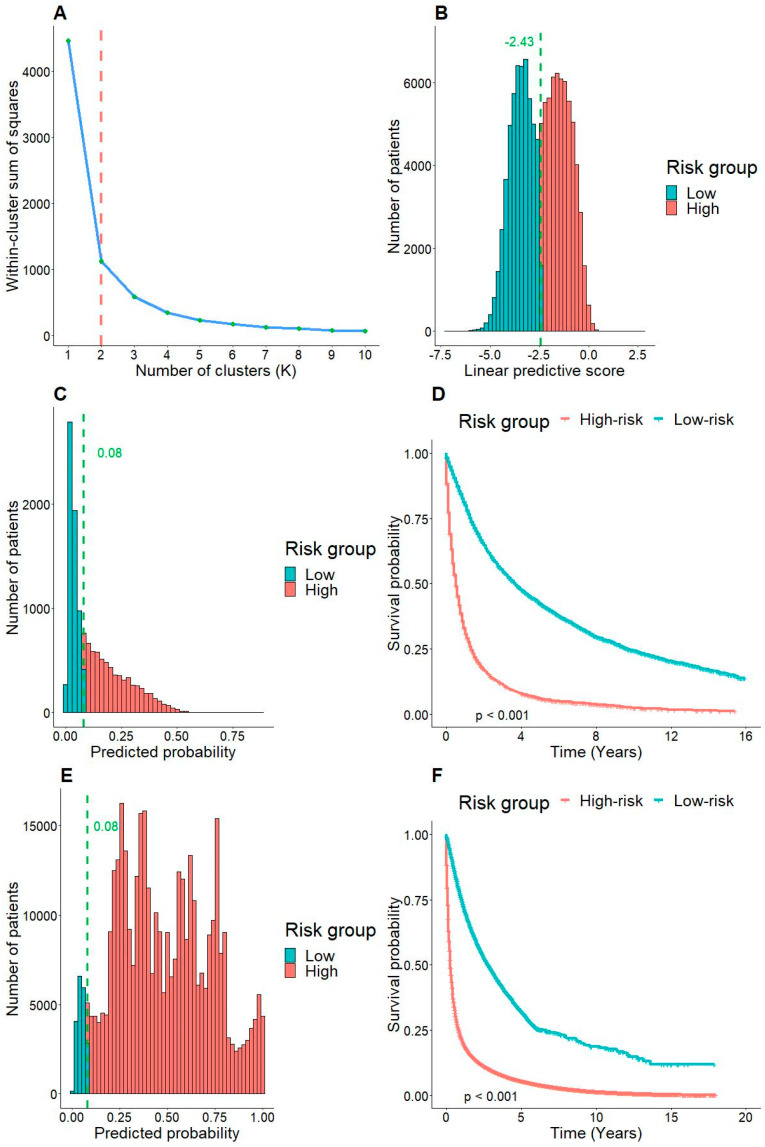
(**A**) The elbow method to find the optimal value of K to cluster the linear predictive score of the model. (**B**) The histogram illustrates the distribution of the predictive score in training data, color coded by the K means clusters. The histograms of predictive scores in validation (**C**) and external validation (**E**) datasets. The Kaplan-Meier curves showed the survival patterns of low-risk and high-risk patients in validation (**D**) and external validation (**F**) cohorts.

We performed a simple K-means clustering algorithm to examine the distribution of the risk values yielded by the model. Figure 4A shows the selection method of K values, using the Silhouette width method. We found that K = 2 was the optimal number of clusters. We next examined the predicted score correlated with each cluster in the training data (Figure 4B). The predictive score was yielded by the formula of the logistic regression equation. Two distinct peaks of predictive scores were observed, which were accurately clustered by the K-means algorithm. The cut-off of risk stratification was −2.43.

We then calculated the predictive scores of each patient in the validation (Figure 4C) and test (Figure 4E) cohorts. The score distributions of validation and test cohorts were distinct, indicating data with different distributions. Low and high-risk patients were classified based on the cut-off determined by the previous analysis. Low-risk patients had the probability of in-hospital mortality less than 8% (Figure 4E). In survival analyses, low-risk and high-risk patients showed a significantly different survival patterns even after discharge. Therefore, our model was not only of in-hospital values but also had predictive power in follow-up observations.

### 3.5. Applications of the Model

**Figure 5:**
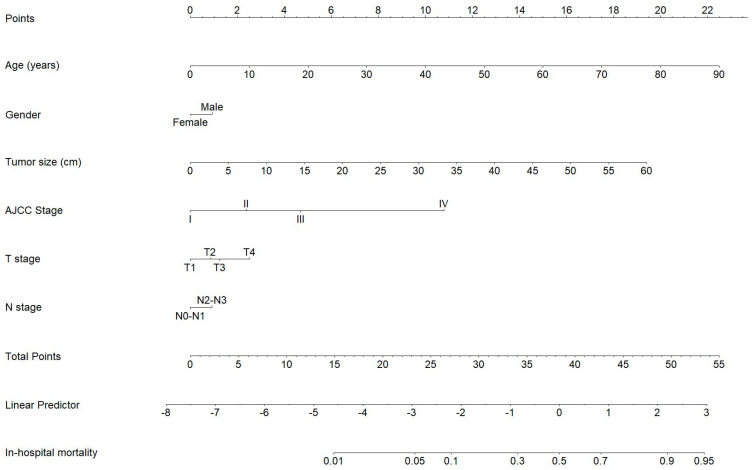
The nomogram illustrates the predictive power of each individual predictor of the model. The uppermost bar presents the score of each variable while the “Total Points” bar is used to estimate the predictive score (“Linear Predictor” bar) and in-hospital mortality probability.

In this section, we created 3 interfaces to use the model, including a static nomogram (Figure 5), a risk table (Table 3), and a dynamic nomogram with a web app. The nomogram visualizes the predictive power of each predictor compared to each other. Each 10 years of age increases the total risk by 2.5 points while each 5 cm of tumor size gives the total score 3 points. Categorical variables including gender (Male—1 point), AJCC stage (stage II—2.5 points; stage III—4.5 points; and stage IV—10.5 points), T stage (T2—0.7 points; T3—1.2 points; T4—2.5 points), and N stage (N2-3—1 point). We translated the risk calculation of the static nomogram into the risk table. These 2 tools have similar scoring system. On the other hand, dynamic nomogram is associated with the shiny web tool that is linked to the author’s account (https://lkhangkv1995.shinyapps.io/LungCancer_In-hospitalMortality-nomogram). This application can yield the predicted probability of in-hospital mortality of the patient of interest as well as its 95%CI. However, it is noteworthy that the model may not yield an accurate prediction when the predicted probability is more than 50%, which can be observed in the calibration plots (Figure 2C,D and Figure 3B).

## 4. Discussion

The model with six fundamental variables including age, gender, tumor size, T, N, and AJCC stages which were initially built to predict the in-hospital mortality of new lung cancer cases resulted in promising applications in prognosis to enhance the quality-of-life of patients diagnosed with this challenging disease. First, in the internal validation extracted from the SEER database, both training and validation cohorts have good performances with an AUC of above 0.75 (Figure 2A,B). In parallel to its counterpart, the external validation also shares a similarly good performance (Figure 3A). However, because the external validation data originated from both SEER data with missing values and TCGA projects, it is assumed that these two sets of data had different data distributions compared to training and validation datasets. Second, the calibration plots also show the fitness of the model with observed in-hospital mortality in the internal validation, particularly in the probability range from 0% to 50%. Although a fair performance with less extent was revealed when it turned to the external one, the probability of in-hospital mortality less than 50% keeps its predictive value. This finding is important for strategic planning for the healthcare of new lung cancer cases, especially in patients with higher survival likelihood. Third, the validation of risk stratification with cut-off −2.43 (Figure 4B) shows a significant difference that is valuable not only in the real-time prediction of in-hospital mortality right after diagnosis (Figure 4C,E) but also in the serial evaluation of OS time (Figure 4D,F). Having said that, while the predicted probability is less than 8% in low-risk patients (Figure 4C,E), two histograms are likely to show visually opposite patterns in terms of the numbers of patients distributed in low- and high-risk due to the heterogeneity of two datasets. It could indicate that there were more high-risk patients based in the external validation group, probably because of late detection, poor-prognosis subtype like SCLC, immunotherapy resistance in NSCLC, etc. Nevertheless, the Kaplan-Meier curves of both cohorts represent significant differences in risk stratification in survival. This reveal has a high possibility to support physicians by predicting in-hospital mortality in the near-term approach and survival in the long run.

Apart from smoking, sex is likely a risk factor to play a specific role in developing lung cancer [14,15]. Age, on the other hand, is usually put in the management context to opt for a cost-effectiveness approach [11,12]. Thus, the combination of the two above demographic variables appears to fill the gap of each factor in prognostic value. Notedly, tumor-directly-related variables such as tumor size, T, N, and AJCC stages in our model are considered together to predict the mortality although there are individual correlations found in the literature regarding lung cancer subtypes [7], frailty [16], or brain metastasis [17]. Although these tumor characteristics possess mutual information, it is important to note that separately scoring each of them comprises a more comprehensive prediction than individual one. For example, tumor size may be related to the T stage, however, full information about tumor size is not available in the T stage. T stage consists of categories of tumor sizes while this system also depends on the local involvement of the tumor [22]. Therefore, the tumor size variable cannot be accessed in its natural form.

From the statistical standpoint, our study shows the predictive value of the training model as the probability varies from 0 to 50% of in-hospital mortality since the initial diagnosis timepoint. Having said that, Shen et al. [17] had a similar approach to ours in terms of the SEER database use to predict death within three months after being diagnosed with lung-cancer-with-brain-metastasis. Nevertheless, instead of using ten variables including metastasis that is likely limited in advanced-stage cases, we only selected six general criteria that can be available not only in wide-range cases with respect to staging, but also accessible in both advanced and low-resource healthcare settings. Despite the fact that our model has less accurate performance with a higher probability of in-hospital mortality as shown in calibration plots, the sensitivity analysis with the external validation added validates the robustness of our model by ζ_Z_ and ζ_T_ values in the 3 contour plots. From these analyses, we confirmed that the model is fairly robust under a different data distribution and an unobserved confounder.

From the clinical standpoint, there are biases and confounding factors can be taken into account. First, in terms of diagnosis, the protocol for the differentiation of primary lung cancer from solitary lung metastasis, especially with unknown primary tumors, is challenging since the lung is considered a frequent metastatic location. Therefore, the model should be applied after the primary lung location is confirmed by careful examination. Second, the policymaking of lung cancer management varies from place to place. Because the model was built based on the SEER database, TCGA-LUAD, and TCGA-LUSC, it partly reflects the outcome of lung cancer management in developed countries. In other words, while the model is helpful to smooth the decision-making among various disease-treatment choices in advanced healthcare settings, it is likely to have less predictive value for patients in a system with fewer resources. Thus, to unveil whether the probability of in-hospital mortality of patients based in developing economies predicted less than 50% is overestimated, further study counting the finance component may need to be conducted. Furthermore, despite the fact “the higher probability, the less predictive value” phenomenon of the model cannot be denied, ultimately the main focus on end-of-life care could be the case in high-risk patients. Likewise, it appears to have an ethic-related controversy surrounding palliative care and dignified death. Hence, a cautious approach should be considered in this group in corresponding to the quality of life. Last, in spite of cutting-edge interventions related to immune-, chemo-radiotherapy, etc., lung cancer is still the leading cause of mortality [1,2,3]. Our model with its robustness could be useful for clinicians to adjust an individualized strategy depending on the real-time risk evaluation at every certain follow-up examination. This helps to improve the survival rate in the landscape of lung cancer.

Regarding applications, we designed three platforms to put the model into practice for quantitative groundwork. The static nomogram (Figure 5) and the web app are friendly to both related parties in the patient-centered therapies and patient education, whereas the risk table (Table 3) can be considered a calculation for physicians to set strategies that are specific to low- and high-risk groups. Indeed, the dynamic nomogram was developed to accurately predict the in-hospital mortality of lung cancer patients which works well in a variety of clinical contexts. In parallel to that, the deployed web app is available and accessible on the internet for global healthcare improvement. Moreover, its visualization platform helps to individualize a particular patient’s risk, the model assists a decision-making intervention as a further step after lung cancer diagnosis for both physicians and patients. In addition, the model can also show the dynamics and circumstances of the predictive values at various time points in the follow-up plan so that the management can be adjusted timely in the long term. On the other hand, we stratify a large number of patients into low- and high-risk groups in the risk table which makes it useful and simple. The cut-off of 26 points for patient differentiation has not only robustness in terms of statistics but also clinical practicality that aids physicians in patient-centered planning. For instance, with respect to the function of rapid response teams (RRT) in the inter-association efforts for survival improvement that have been emphasized in prior studies [23,24], our model is advantageous to leverage their role by focusing on high-risk groups in terms of end-of-life care enhancement. Consequently, in order to regulate possible delays, healthcare institutes can efficiently implement a system that pays attention to those vulnerable patients so that RRT can have timely activation. Having said that, in terms of the applications of machine learning in real-life clinical settings, it seems that they are in the early stages of putting theory into practice despite a tremendous amount of potential research. For instance, non-invasive imaging with its important role in diagnosis and follow-up is suited to become a pioneering bridge between academic and clinical [25]. Hence, our machine-learning applications in lung cancer are promising but require a cautious approach to the traditional clinical.

The study has its limitations. First, the data extracted from SEER and TCGA-LUAD, and TCGA-LUSC with different regimes made them heterogeneous. Since metastasis diagnosis has a possibility to be missed in new primary cancer diagnoses, we hope to minimize this bias by excluding the metastasis variable. However, a pilot validation in clinical may be necessary to examine the model for a comprehensive application. Second, as mentioned in the Results section, the model turned out to have a less accurate value with the probability of an in-hospital mortality rate above 50%. This limitation infers the important role of end-of-life care regardless of the survival rate. Finally, the population-based design of the study without subtype classification of NSCLC and SCLC missed the molecular insight reasoning for immunotherapeutic that has played a game changer for the survival rate improvement in recent decades, especially in countries with advanced healthcare systems. Thus, to figure out how the high- and low-risk patient discrimination are correlated to the subtypes of lung cancer like NSCLC and SCLC, future studies can possibly include that variable for an optimal model.

## 5. Conclusions

To conclude, our machine learning-based model trained and validated shows a high predictive value for the in-hospital mortality of new lung cancer patients, especially in the probability of less than 50%. Three applications including the static nomogram, the web app, and the risk table are helpful and accurate for risk stratification. Indeed, as more and more novel therapies for lung cancer treatment are accessible, more and more new prognosis-related questions need to be addressed. Our applications, especially the web app, are available on the internet and applicable to a wide range of healthcare settings in the world. Additionally, they can aid physicians to consult not only patients with “low-risk” in-hospital mortality for strategic planning but also those with “high-risk” one for end-of-life care. In combination with the molecular investigation, the model with its robustness can assist clinical beyond that for further interventions.

## Figures and Tables

**Table 1:** Patient’s characteristics of training, validation, and test datasets.

Variable	Train (*n* = 115,145)	Validation (*n* = 13,017)	Test (*n* = 395,797)	*p*-Value
Age (years)	71 (2–85)	71 (19–85)	69 (0–90.1)	<0.001
Gender				<0.001
Women	56,592 (49.1%)	6411 (49.3%)	169,427 (42.8%)	
Men	58,553 (50.9%)	6606 (50.7%)	226,370 (57.2%)	
Race				<0.001
AIAN ^1^	657 (0.6%)	72 (0.6%)	1757 (0.4%)	
API ^2^	11,176 (9.7%)	1262 (9.7%)	28,408 (7.2%)	
Black	9882 (8.6%)	1107 (8.5%)	28,956 (7.3%)	
White	93,237 (81.0%)	10,557 (85.0%)	336,046 (84.9%)	
Not reported	193 (0.2%)	19 (0.1%)	630 (0.2%)	
Tumor size (cm)	3.4 (0–60.0)	3.5 (0.1–4.9)	3.4 (0–60.0)	0.494
AJCC stage				<0.001
Stage I	35,057 (30.4%)	3968 (30.5%)	31,265 (24.0%)	
Stage II	9976 (8.7%)	1123 (8.6%)	9246 (7.1%)	
Stage III	25,315 (22.0%)	2876 (22.1%)	27,869 (21.4%)	
Stage IV	44,797 (38.9%)	5050 (38.0%)	62,021 (47.6%)	
T stage				<0.001
T1	35,394 (30.7%)	3957 (30.4%)	31,465 (26.2%)	
T2	35,094 (30.5%)	4061 (31.2%)	34,654 (28.8%)	
T3	16,047 (13.9%)	1774 (13.6%)	16,704 (13.9%)	
T4	28,610 (24.8%)	3225 (24.8%)	37,345 (31.1%)	
N stage				<0.001
N0	54,856 (47.6%)	6181 (47.5%)	54,623 (43.7%)	
N1	10,863 (9.4%)	1209 (9.3%)	11,669 (9.3%)	
N2	36,268 (31.5%)	4167 (32.0%)	42,436 (34.0%)	
N3	13,158 (11.4%)	1460 (11.2%)	16,188 (13.0%)	
M stage				<0.001
M0	70,348 (61.1%)	7967 (61.2%)	75,528 (55.0%)	
M1	44,797 (38.9%)	5050 (38.8%)	61,740 (45.0%)	
Surgery				<0.001
No	80,031 (69.5%)	9076 (69.7%)	169,300 (42.8%)	
Yes	35,114 (30.5%)	3941 (30.3%)	226,497 (57.2%)	
In-hospital mortality				<0.001
No	100,360 (87.2%)	11,302 (86.8%)	311,582 (80.6%)	
Yes	14,785 (12.8%)	1715 (13.2%)	74,905 (19.4%)	
Survival time (month)	12 (0–191)	12 (0–191)	8 (0–539)	0.494

^1^ AIAN: American Indian/Alaska Native; ^2^ API: Asian or Pacific Islander.

**Table 2:** Univariate and multivariate logistic regression analyses of in-hospital mortality in the training dataset.

Variable	Univariate			Multivariate		
	OR ^1^	95%CI ^2^	*p*-Value	OR	95%CI	*p*-Value
Age (years)	1.04	1.04–1.04	<0.001	1.05	1.05–1.05	<0.001
Gender						
Female	1.00					
Male	1.30	1.26–1.35	<0.001	1.20	1.16–1.25	<0.001
Race						
AIAN *	1.00					
Asian	1.12	0.87–1.45	0.390			
Black	1.28	0.99–1.66	0.056			
White	0.62	0.33–1.14	0.122			
Not reported	1.27	0.99–1.63	0.059			
Tumor size (cm)	1.16	1.15–1.16	<0.001	1.07	1.06–1.07	<0.001
T stage						
T1	1.00			1.00		
T2	2.15	2.04–2.28	<0.001	1.18	1.10–1.25	<0.001
T3	3.39	3.19–3.61	<0.001	1.28	1.19–1.37	<0.001
T4	5.09	4.83–5.37	<0.001	1.64	1.54–1.76	<0.001
N stage						
N0	1.00			1.00		
N1	1.70	1.59–1.81	<0.001	1.00	0.93–1.08	0.950
N2	3.00	2.88–3.12	<0.001	1.23	1.17–1.30	<0.001
N3	2.87	2.72–3.03	<0.001	1.09	1.03–1.16	0.005
M stage						
M0	1.00			n/a	n/a	
M1	5.58	5.36–5.80	<0.001	n/a	n/a	
AJCC stages						
I	1.00			1.00		
II	1.99	1.78–2.23	<0.001	1.59	1.41–1.80	<0.001
III	4.07	3.77–4.40	<0.001	2.48	2.26–2.72	<0.001
IV	12.25	11.43–13.14	<0.001	8.20	7.58–8.97	<0.001

* AIAN: American Indian/Alaska Native; ^1^ OR: odds ratio; ^2^ 95%CI: 95% confidence interval.

**Table 3:** Risk table of in-hospital mortality of lung cancer patients.

Variable	Score
Age (years)	2.5 scores per 10 years
Gender	
Women	0
Men	1
Tumor size (cm)	3 scores per 10 cm
AJCC stage	
Stage I	0
Stage II	2.5
Stage III	4.5
Stage IV	10.5
T stage	
T1	0
T2	0.7
T3	1.2
T4	2.5
N stage	
N0-N1	0
N2-N3	1
**Risk group**	**Total score**
Low-risk	<26
High-risk	≥26

## Data Availability

The datasets generated and analyzed during the current study are available in the Surveillance, Epidemiology, and End Results (SEER) and The Cancer Genome Atlas (TCGA), https://seer.cancer.gov and https://www.cancer.gov/ccg/research/genome-sequencing/tcga accessed on 1st December 2022.

## Supplementary Materials

The following supporting information can be downloaded at: https://www.mdpi.com/article/10.3390/arm91040025/s1, Figure S1: Pie Charts of Data Availability in External Test Cohort; Table S1: Univariate and multivariate logistic regression analyses of in-hospital mortality in training dataset; Table S2. Variable-specific train and test errors of HI-VAE.
