# Supplementary material for: A Machine Learning-Based Model to Predict In-Hospital Mortality of Lung Cancer Patients: A Population-Based Study of 523,959 Cases
Source: Adv Respir Med. 2023 Aug 9;91(4):310–23. doi: 10.3390/arm91040025 (PMC10451707; doi:10.3390/arm91040025)
Supplement: Supplementary file 1 [file arm-91-00025-s001.zip › arm-2523116-supplementary.pdf]

Table S1. Univariate and multivariate logistic regression analyses of in-hospital mortality in training dataset.

	Univariate			Multivariate		
**Variable**	**OR[1]**	**95%CI[2]**	**p-value**	**OR**	**95%CI**	**p-value**
Age (years)	1.04	1.04 - 1.04	<0.001	1.05	1.05 - 1.05	<0.001
Gender						
Female	1.00					
Male	1.30	1.26 - 1.35	<0.001	1.20	1.15 - 1.24	<0.001
Race						
AIAN*	1.00					
Asian	1.12	0.87 - 1.45	0.390			
Black	1.28	0.99 -1.66	0.056			
White	0.62	0.33 - 1.14	0.122			
Not reported	1.27	0.99 - 1.63	0.059			
Tumor size (cm)	1.16	1.15 - 1.16	<0.001	1.07	1.06 - 1.07	<0.001
T stage						
T1	1.00			1.00		
T2	2.15	2.04 - 2.28	<0.001	1.18	1.11 - 1.26	<0.001
T3	3.39	3.19 - 3.61	<0.001	1.27	1.18 - 1.37	<0.001
T4	5.09	4.83 - 5.37	<0.001	1.64	1.54 - 1.75	<0.001
N stage						
N0-N1	1.00			1.00		
N2-N3	2.67	2.58 - 2.77	<0.001	1.19	1.14 - 1.25	<0.001
M stage						
M0	1.00			n/a	n/a	
M1	5.58	5.36 - 5.80	<0.001	n/a	n/a	
AJCC stages						
I	1.00			1.00		
II	1.99	1.78 - 2.23	<0.001	1.59	1.42 - 1.79	<0.001
III	4.07	3.77 - 4.40	<0.001	2.51	2.29 - 2.74	<0.001
IV	12.25	11.43 - 13.14	<0.001	8.26	7.61 - 8.96	<0.001

*AIAN: American Indian/Alaska Native; [1]OR: odds ratio; [2]95%CI: 95% confidence interval.

Table S2. Variable-specific train and test errors of HI-VAE.

Variable	Train error	Test error	p-value
Age	0.056 (±0.001)	0.128 (±0.000)	< 0.001
Gender	0.167 (±0.002)	0.431 (±0.001)	< 0.001
Tumor size (cm)	0.050 (±0.000)	0.045 (±0.000)	< 0.001
T stage	0.198 (±0.003)	0.657 (±0.016)	< 0.001
N stage	0.048 (±0.004)	0.096 (±0.000)	< 0.001
M stage	0.050 (±0.008)	0.568 (±0.004)	< 0.001
AJCC stage	0.047 (±0.003)	0.598 (±0.001)	< 0.001
In-hospital mortality	0.069 (±0.003)	0.172 (±0.000)	< 0.001

There are three different types of errors, depending on whether the variable is continuous (age, tumor size), categorical (gender, N, M, and in-hospital mortality), or ordinal (T and AJCC stage). The types of errors are normalized root mean square error, accuracy error, and displacement error, respectively. Further details of these errors can be found in the original study [20].

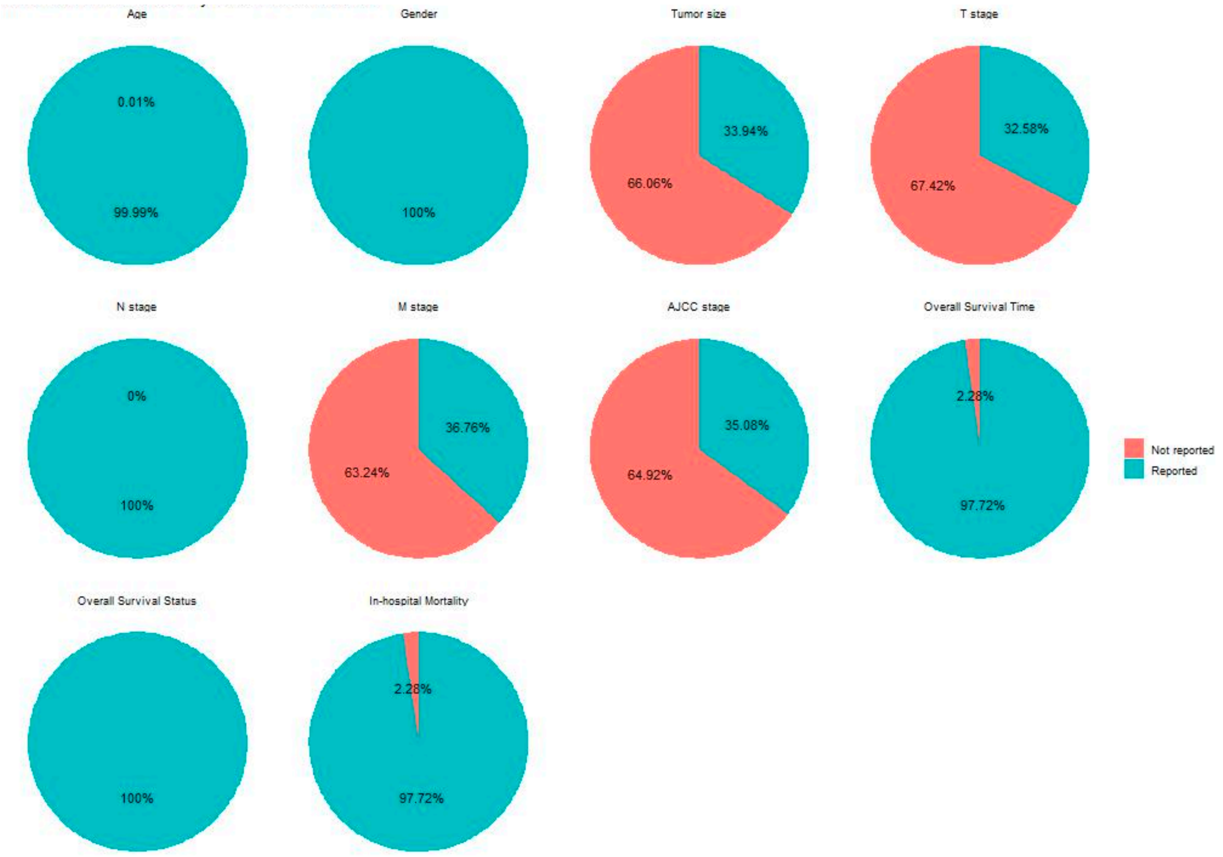

Figure S1. Pie Charts of Data Availability in External Test Cohort.
